# Decomposition of life expectancy differentials with (and without) conditions by educational attainment for major groups of causes in contemporary Spain: where is the advantage?

**DOI:** 10.1186/s41118-024-00220-5

**Published:** 2024-07-16

**Authors:** Octavio Bramajo, Pilar Zueras, Elisenda Rentería, Iñaki Permanyer

**Affiliations:** 1 Universitat Autònoma de Barcelona, Bellaterra, Spain; 2 Centre d’Estudis Demogràfics, Bellaterra, Spain; 3 University of Texas Medical Branch, Galveston, USA; 4 Institute for Social and Economic Research, University of Essex, Essex, UK; 5 ICREA-Institució Catalana de Recerca i Estudis Avançats, Barcelona, Spain

**Keywords:** Aging, Social inequalities, Spain, Morbidity, Mortality

## Abstract

**Introduction:**

Healthy life expectancy is higher among individuals with higher socioeconomic standing. However, it is unclear whether such advantage is attributable to longer (i.e., mortality advantage) or to healthier (morbidity advantage) lifespans across different health conditions.

**Objective:**

Estimate the contribution of mortality and morbidity components in differences in condition-free life expectancies (CFLE) and life expectancy with conditions (LEWC) for five major groups of conditions by sex and educational attainment, instead of using a global indicator of morbidity.

**Methods:**

Using the Sullivan Method, we computed remaining life expectancies at age 40 and 65, CFLE, and LEWC and applied a stepwise decomposition technique, using national health surveys along with mortality data, in a cross-sectional analysis.

**Results:**

An educational gradient was present in almost all conditions, with different intensities. For females, morbidity was the main contributor to educational differences in health expectancies, but mainly in the older age groups. For males, the drivers behind higher health expectancies for high-educated males were evenly distributed across mortality and morbidity between ages 40 and 65, but after that, the mortality gradient vanished between high-educated and middle-educated individuals.

**Discussion:**

The changing contribution of the mortality and morbidity gradient for different conditions across age-groups brings evidence to adequately plan health policies to mitigate health gaps and improve quality of life of the populations in a lower social standing.

## Introduction, research questions, and objective

A deeper understanding of the dynamics of socioeconomic disparities in the health burden of a population is key to identifying and addressing inequalities in health. Despite the substantial increase in life expectancy across the globe due to medical breakthroughs, nutritional improvements, better sanitation, and the adoption of healthier behaviors (Deaton, 2005; Fogel & Costa, 1997), this process has shown important disparities across different socioeconomic groups (i.e., Hummer & Lariscy, 2011; Link & Phelan, 1995; Montez et al., 2012). Spain has been no exception to such a trend and is currently one of the countries with the highest longevity in the world, although these improvements tend to favor the wealthier or more educated individuals in the population (Permanyer et al., 2018; Regidor et al., 2015, 2016). The so-called cardiovascular revolution (Vallin & Meslé, 2004) entailed a transition from high cardiovascular disease mortality to increased survival attributed to those conditions in Western countries. This has led to a great improvement in adult mortality rates, setting the tone for a stronger emphasis on the quality of survival and the prevalence of chronic conditions and ailments, both lethal and non-lethal.

The relationship between having a longer lifespan and a healthier lifespan (expressed in terms of disability, morbidity, and prevalence of a disease or any other given indicator) differs by socioeconomic status. However, these processes are not entirely understood yet. While several studies have evidenced that higher-educated individuals have longer and healthier lifespans in Spain (Solé-Auró et al., 2020, 2022; Gumà et al., 2019, among others), the demographic drivers behind such differences are unknown. Are healthier lifespans just the result of lower overall morbidity? Do these mechanisms operate similarly across socioeconomic status, sex, and health conditions? The objective of this paper is to identify whether the advantage of healthy life expectancy among higher educated people in Spain is due to a longer lifespan (reflecting mortality advantages), a healthier lifespan (morbidity advantages), or a combination of both (double advantage). We use educational attainment as a proxy for socioeconomic status and analyze a set of common conditions that affect the overall health of the population across their life course.

## Background

Different theories have been proposed to analyze the dynamics of the relationship between mortality and morbidity, without a single vision imposing over the others (Gruenberg, 1977; Fries, 1984; Manton et al., 2006; Myers and Manton, 1984). This relationship between the time spent with good health and overall longevity, however, is not as straightforward as it could be expected when looking into differences by sex and socioeconomic status. In what is known as the health-survival paradox (Di Lego et al., 2020; Van Oyen et al., 2013), females have a longer life expectancy than males, but they also spend a larger proportion of their lives in poor health in terms of the prevalence of chronic conditions, morbidity, and disability.

In addition to gender differences, there is also a socioeconomic gradient: individuals with higher socioeconomic status live longer and healthier lives when compared to their counterparts with a lower socioeconomic status. Theory of the *Fundamental Cause* proposes a dynamic relationship between socioeconomic status and health, suggesting that there is a clear link between lower socioeconomic status and many negative health outcomes that can manifest themselves through a variety of everchanging mechanisms over the life course, but also over time (Link & Phelan, 1995). Better education tends to be correlated with having a higher socioeconomic status, which in turn is associated with having more opportunities to make healthier life choices, as well as greater capability to procure goods and services to maintain good health and lesser exposure to risky behaviors and environments (Cutler et al., 2006; Marmot, 2005). Previous research highlighting this link has been conducted in many countries, such as the US (i.e., Hayward et al., 2015; Montez et al., 2012; Nusselder et al., 2005), Europe (i.e., Mackenbach, 2012; van Raalte et al., 2014; Zazueta-Borboa et al., 2023) and Latin America (i.e., Beltrán-Sánchez & Andrade, 2013; Bramajo & Grushka, 2020; Sandoval & Turra, 2015), to name a few examples.

In Spain, people with higher socioeconomic status also have healthier lives (Cambois et al., 2019; Gumà et al., 2019; Solé-Auró et al., 2020, 2022). This has also been found in specific studies of certain regions, such as Catalonia (Solé-Auró & Alcañiz, 2015; Walter et al., 2016). More specifically, whether using global indicators of health or broad ad hoc definitions of poor health, previous studies have estimated the educational gap in health expectancies in Spain (Cambois et al., 2016; Solé-Auró & Alcañiz, 2016; Solé-Auró et al., 2020, 2022). In these studies, aggregate indexes (which we refer to here broadly as health expectancies) such as healthy life expectancy (HLE) or condition-free life expectancy (CFLE) have been computed to determine the total health burden in a population over the lifespan. However, the drivers of this health advantage are unclear: given that health expectancy is not independent of life expectancy (van Raalte & Nepomuceno, 2020), it is not obvious to what extent the advantage of the higher educated in Spain (in terms of health expectancy) is the result of lower mortality or lower morbidity. This is relevant because interventions and policies aimed at reducing health inequalities require accurate measurement and estimation. Determining the extent to which disparities in the time lived with a condition is the result of a longer/shorter or a healthier/unhealthier life expectancy is important for a more comprehensive assessment of the overall impact and implications of these disparities.

Furthermore, the health expectancies estimated in the aforementioned studies were generally computed using aggregate, catch-all indicators, such as the Global Activity Limitation Indicator, better known as GALI (Van Oyen et al., 2018), or the presence of limitations in instrumental activities of daily living or IADL (Crimmins et al., 2009; Manton et al., 2006). Few studies have estimated gaps in health expectancies for different major groups of diseases in other countries such as the US or Belgium (Nusselder & Looman, 2004; Nusselder et al., 2005; Yokota et al., 2019). In Spain, previous studies analyzing disparities in health expectancies have considered different definitions of poor health (Solé-Auró & Alcañiz, 2015; Solé-Auró et al., 2022; Walter et al., 2016) or the presence of one or multiple diseases to establish a poor health status, rather than considering a cause-specific analysis (Cleries et al., 2009; Solé-Auró et al., 2020; Zueras & Rentería, 2020).

However, not every condition or group of conditions has the same socioeconomic and sex gradient when addressing health disparities. For instance, it has been widely acknowledged that mood and mental disorders such as anxiety or major depression affect more females than males (Blazer & Hybels, 2005; Cardila et al., 2015; Gispert et al., 1998). The same occurs with other conditions such as coronary heart disease (a cardiovascular condition), arterial hypertension, or diabetes mellitus (Cordero et al., 2009; Gao et al., 2019; Maas & Appelman, 2010; Mauvais-Jarvis, 2018) where males have a higher prevalence. While some of these conditions are non-lethal per se, they may be associated with multiple negative health outcomes due to their impact on quality of life, including death. Moreover, different conditions affect populations with particular intensity at different stages of life.

Therefore, examining the education gap separately for each cause can provide a broader perspective on how health inequalities operate in Spain. As Nusselder and Looman (2004) point out, knowing which age groups and which diseases contribute the most to differences in population health helps to identify drivers and determinants of such differences is helpful to produce policies to eventually reduce them. Given these gaps in the literature, we wanted to determine whether, for different diseases or health conditions, the health expectancy advantage of the higher-educated individuals (or the disadvantage of their lower-educated counterparts) in Spain was due to a mortality advantage, a morbidity advantage, or both. We did this by considering five major groups of conditions, separately by sex.

## Data source and methods

### Data sources

We relied on a combination of mortality and morbidity data sources to produce the necessary estimates for this study in a given period. For mortality data, we used both mortality data and the population exposures provided by the National Institute of Statistics (“Instituto Nacional de Estadística” in Spanish, also known by its acronym INE) for the 2014–2016 period. INE used a matching algorithm linking registered deaths to population databases, including censuses, municipal population registers, the Ministry of Education, and the Public State Employment Service, to obtain and provide overall death counts according to educational attainment. The INE also provided the total estimates of population by sex, age, and educational attainment (in a series of different categorical values) in Spain for the analyzed years. This combination of sources has been used successfully in previous studies in the past (e.g., Permanyer et al., 2018).

To obtain the diagnosed prevalence of the selected chronic conditions we used the Spanish National Health Survey (or ENSE, its acronym in Spanish), which has been conducted periodically from 1987 to 2017, and the Spanish data from the European Health Interview Survey (EHIS). This information is freely available from the Spanish Ministry of Health. Those surveys are cross-sectional, multiple wave representative studies covering the general health situation in Spain at the national and regional levels (Autonomous Communities, the main administrative unit in Spain) among the non-institutionalized population. Hereafter, prevalence refers to the prevalence in the non-institutionalized population. The ENSE and the EHIS are both conducted by the INE. However, the latter is coordinated by Eurostat, with an adapted questionnaire to allow the main indicators of both surveys to be comparable over time. The questionnaires were administered to individuals aged 15 and over living in private households across Spain. Questionnaires collect sociodemographic information and health conditions and determinants of the population, including health service use and lifestyles among others.

We worked with five major groups of chronic conditions for our estimations, based on their larger prevalence across the Spanish population: cardiovascular diseases (CVD) (‘High Cholesterol’, ‘Myocardial Infarction’, ‘Stroke’, and ‘other Heart Diseases’); diabetes; arterial hypertension (for practical reasons, we will refer to it simply as ‘Hypertension’); mental disorders (‘Depression’, ‘Anxiety’ and ‘other Mental Disorders’); and respiratory conditions (‘Asthma’ and ‘Chronic Obstructive Pulmonary Disease’, COPD). To consider if the respondent had been diagnosed with the selected condition, we used a criterion similar to the one adopted by Zueras and Rentería (2020). This criterion considered that the respondent had to have the specific health condition in the last 12 months and it had to be diagnosed by a physician as well (however, the condition is self-reported in both situations). The details of the questionnaires for each category can be found in the Appendix in Table 6. As discussed by Zueras and Rentería (2020) uneven healthcare access may result in problematic reporting on health conditions. However, Spain has a low percentage of unmet medical needs in terms of diagnosis or treatment (OECD, 2017), so we expect that underreporting errors may be small.

To make the data as robust as possible, we pooled the 2012 and 2017 ENSE survey waves and the 2014 EHIS survey results, assuming that the prevalence estimates were the average for the period analyzed and that abrupt changes in prevalence and diagnosis from year to year are unlikely, given the inertial nature of demographic change. The population structure of the surveys is almost identical, and representative of the non-institutionalized population at a national level. Furthermore, we pooled the surveys to obtain confidence intervals in health expectancies that otherwise might be too wide and potentially unreliable (since the standard error would be larger in a smaller sample). Complementarily, we also pooled mortality data to make a reasonable comparison, centered on 2015.

Educational level was split into three groups: individuals with lower educational attainment (who have completed at most the first cycle of secondary education, which corresponds to level 2 in the normalized ISCED-2011 classification), individuals with mid education (levels 3 and 4 in ISCED-2011 classification) and individuals with higher educational attainment (ISCED-2011 level 5, having completed short-cycle tertiary education, and above). Age 40 was defined as the starting point for the estimates because the prevalence of most conditions was negligible before that age.

### Methods

The life table was top truncated at age 85 (given that sometimes prevalence is hard to estimate beyond that point without some strong modeling assumptions). As a usual practice, we smoothed the death rates in 5-year age groups by using a one-dimensional Poisson P-spline, available in the *MortalitySmooth* package (Camarda, 2012).

First, as an exploratory analysis, we plotted age-specific death rates and age-specific prevalence for each condition, separately by sex and education. With these components, we produced a set of life tables (separately by sex and educational attainment) to estimate the remaining life expectancy at age 40 (Preston et al., 2001). However, after some preliminary testing, we produced estimates for age 65 as well to conduct more exhaustive analyses. The five-age group prevalence estimates of each chronic condition, by sex and education, were applied to the corresponding life table to obtain the condition-free life expectancies (CFLE) at age 40, using the Sullivan method, whose purpose is to compute the proportion of time lived with or without a given condition (Sullivan, 1971) and is the most widely used method for cross-sectional data. We also estimated the life expectancy with the condition (LEWC), representing the number of years lived with the examined condition, which is simply the difference between LE and CFLE. We calculated 95% confidence intervals (CI) for the CFLE and LEWC using the suggested procedure by Jagger et al. (2014). However, given the large population size used for mortality data (a minimum exposure above 15 million for each combination of sex and educational attainment), the range of lower and upper estimates for overall life expectancy was minimal, and as a result, we only presented the point estimate. By using the Sullivan method, we are assuming that the mortality information is constant for all educational groups, given that we do not have cause-specific mortality by education and, therefore, we cannot establish an education gradient to condition lethality, if any. Although this is a limitation of this study, as described below, we believe that this study contributes to a deeper understanding of socioeconomic inequalities in health expectancies.

Second, we establish two components to compute differences between CFLE and LEWC: differences based on mortality (longevity) and differences based on morbidity (prevalence). To establish the contribution of each component, we relied on a generalized decomposition technique (Nusselder & Looman, 2004; van Raalte & Nepomuceno, 2020). The decomposition can be applied to different moments for a given population (considering compositional changes over time), or to two populations at a single moment (considering different compositions between compared groups). Such techniques identify the contribution of morbidity and mortality components (Andreev et al., 2002; van Raalte & Nepomuceno, 2020), allowing us to appropriately determine the role of each one in the obtained difference in two health expectancies, and which age groups are the ones that concentrate such contributions.

In this case, we relied on the decomposition algorithm of stepwise replacement first described by Andreev et al. (2002). This technique changes the selected components (in this case, mortality and morbidity) sequentially and recalculates the index function to obtain the contribution of each parameter to the aggregate result. Moreover, this procedure allows us to perform an age-specific decomposition for the differences in health expectancy into mortality and morbidity components. Such a procedure is already incorporated in the *DemoDecomp* package built by Riffe (2018).

The change in the number of years lived without the selected condition (CFLE) between populations 1 and 2 can be broken down as the sum of a mortality (longevity) component (MORT) and a morbidity (prevalence) component (MORB): that is: CFLE_2_ − CFLE_1_ = MORT + MORB. In our case, populations 1 and 2 would represent the corresponding subpopulations by educational attainment. The final form of the technique is presented in Andreev et al. (2002).

Third, we present the results of these decompositions considering each educational gap. This is the difference between the middle-educated minus the low-educated, and the difference between the high-educated minus the middle-educated group, while the gap between the high-educated and the low-educated would simply be the sum of these two separate gaps. In addition, the value of each component is equal to the sum of their respective age-specific contributions, facilitating an age-specific decomposition.

This was done for each of the five conditions analyzed, for both males and females, and considering the gaps in CFLE and LEWC. Given that CFLE and LEWC are complementary measures, the contribution of morbidity to a given difference in CFLEs should be the opposite of the contribution of morbidity to the corresponding difference in LEWCs (since the prevalence used for one indicator is the complement of the other). For practical reasons, we present only the average decomposition differentials to visualize the contribution of each component. All visualizations in this study were done with ggplot2 package (Wickham, 2015), freely available in R software. All estimations, tables and figures are authors’ calculations.

## Results

### Exploratory analyses

Firstly, we performed an exploration of the data sources (health surveys, aggregate mortality data, and population exposures). Table 1 presents the composition of the sample by sex, age, and educational attainment and the prevalence rates of the conditions analyzed, as well as the distribution of the mortality data by educational level and population exposures. The pooled sample included 47,024 observations with 55.3% identified as female, and a mean age of 59.7 years. Among the conditions studied, hypertension and CVD had a similar prevalence, of 31.8% and 32%, respectively. Mental disorders followed them with a prevalence of 19.1%, and diabetes a 12%. Respiratory conditions had the lowest prevalence, with 8.2%. The mortality data presented almost a million deaths, with the majority corresponding to individuals of the low education group, while the population exposures involved more than 61 million person-years (given that multiple years are pooled).

**Table 1:** Descriptive statistics for numeric variables of people aged 40 years or more

Variable		
Number of pooled observations in surveys (cases)	47,024	
Share of males (%)	44.7	
Age (mean)	59.7	
CVD (%)	32.0	
Diabetes (%)	12.0	
Hypertension (%)	31.8	
Mental disorders (%)	19.1	
Respiratory (%)	8.2	
Low education (average %)	17.6	
Middle education (average %)	56.0	
High education (average %)	26.2	
Number of observations in mortality data (deaths)	942,234	
Share of deaths by low education (%)	83.2	
Share of deaths by middle education (%)	8.9	
Share of deaths by high education (%)	7.9	
Population exposures (in person-years)	61,076,657	
Share of population exposure by low education (%)	57.5	
Share of population exposure by middle education (%)	17.4	
Share of population exposure by high education (%)	25.1	

Source: author’s calculations based on INE, ENSE and EHIS

**Fig. 1:**
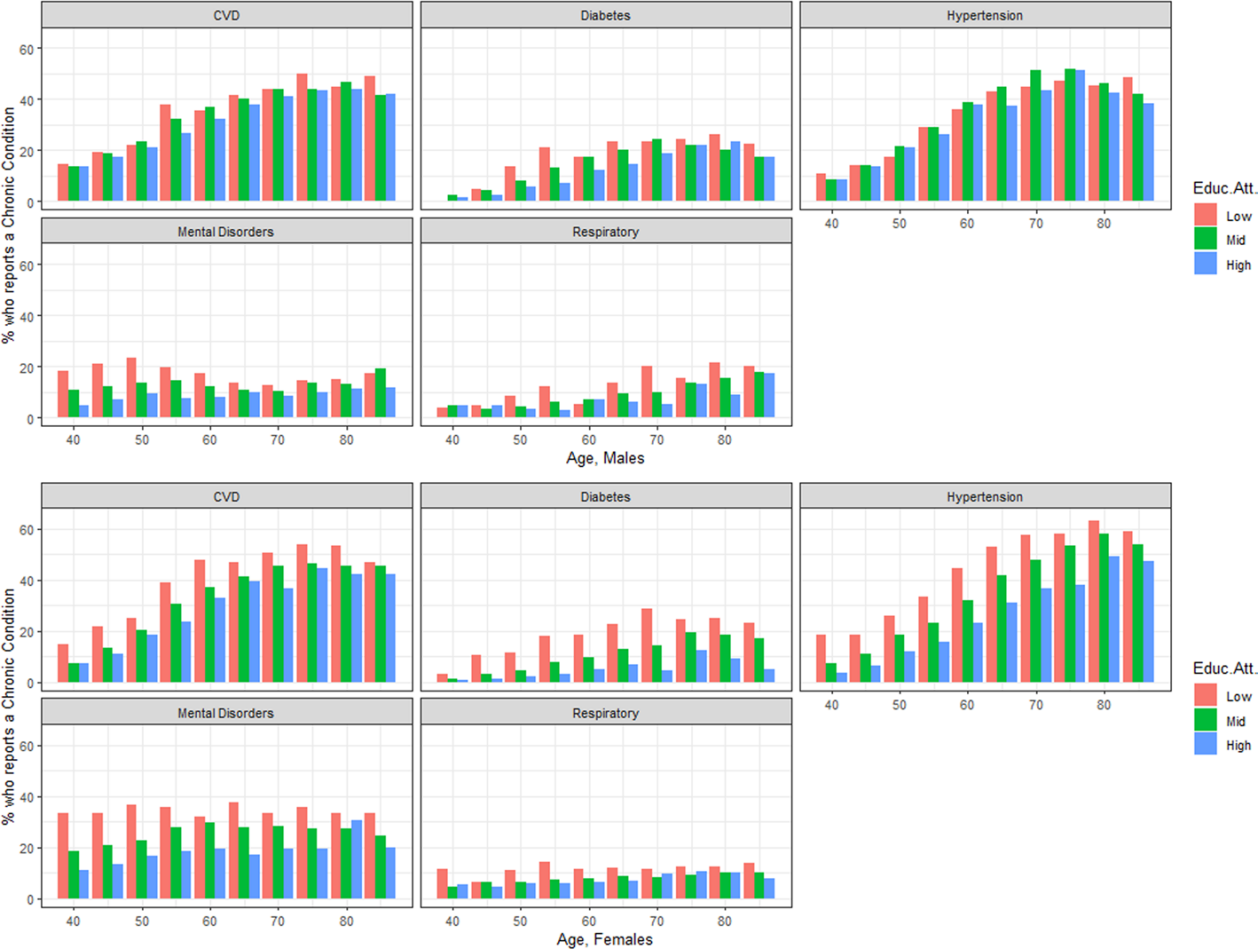
Age-specific prevalence of groups of conditions by educational attainment for ages 40 to 85+, separately by sex. Spain, 2012–2017 (Source: author’s calculations based on ENSE and EHIS)

Figure 1 shows the age-specific prevalence of each group of conditions separately by sex and by educational attainment across the reported conditions. For males, educational differences in prevalence are small for most of the conditions. Divergences in respiratory conditions begin to be notorious from the age of 60 and persist thereafter. Diabetes presents small but persistent gradients across the life course. Mental disorders present the most marked differences in education at younger age groups, before converging at older ages. For females, the educational gradient is more pronounced across the life course. Inequalities are marked and persistent for almost all conditions, with the sole exception of the respiratory ones. Narrow differences between the middle and high educational groups are also found for CVD.

**Fig. 2:**
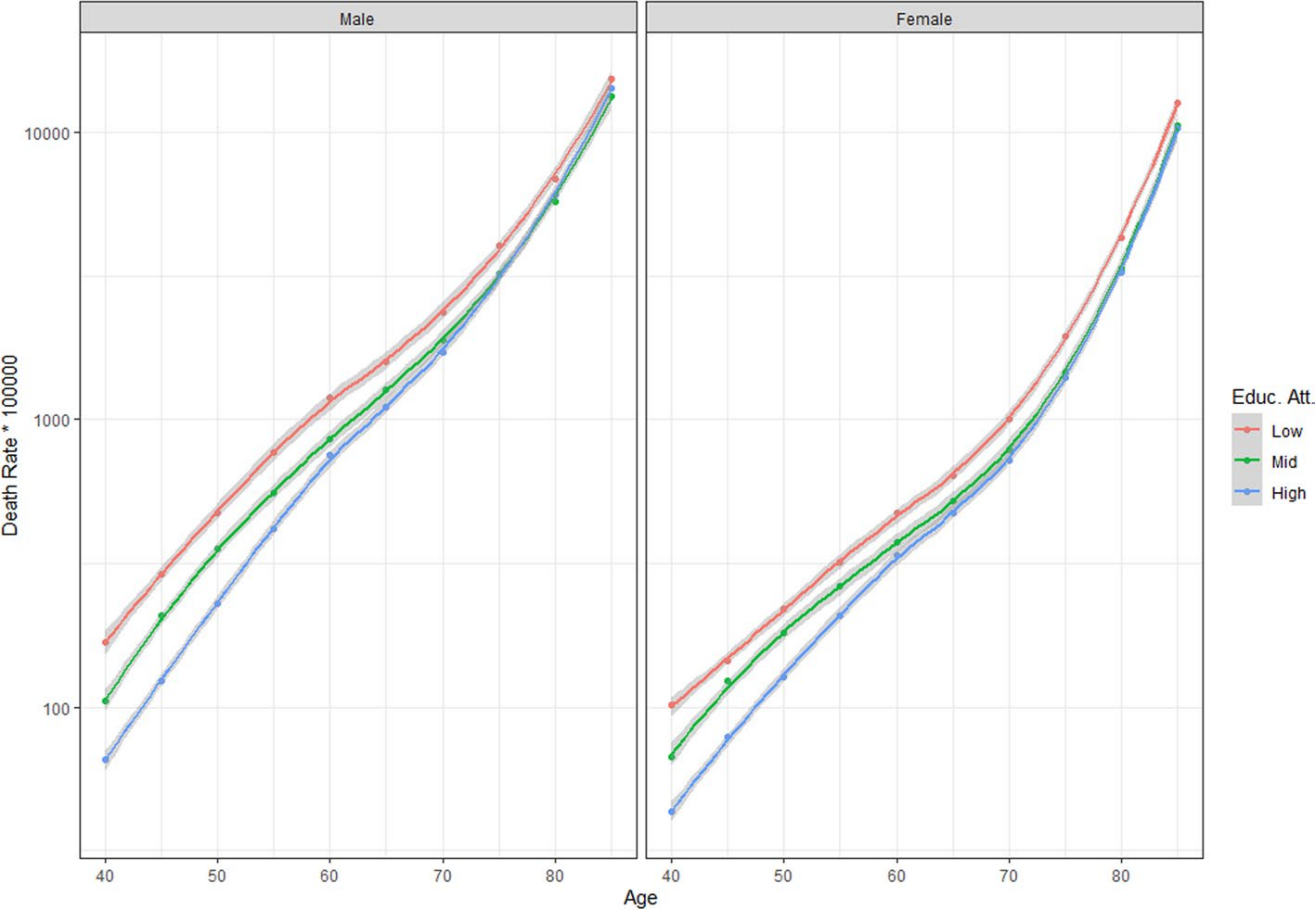
Death rates (*100,000) by educational attainment for ages 40 to 85 + , Spain, 2012–2017 (Source: author’s calculations based on INE)

Figure 2 presents the age-specific death rates by educational attainment. While it is evident that low-educated individuals present the highest mortality rate, the initial advantage of high-educated individuals over the middle education group disappears around age 65 for females and age 70 for males. The figure shows a convergence afterwards, but the low education group presents higher death rates at all age groups.

### Life expectancy, condition-free life expectancy (CFLE) and life expectancy with condition (LEWC) at ages 40 and 65

Table 2 presents estimates of the remaining life expectancy at age 40 (LE40) and the corresponding CFLE and LEWC computed with a 95% confidence interval (CI). This was done for the five selected conditions, separately by sex and educational attainment. Table 3 presents the estimates for the same indicators at age 65 (LE65). Results show that females had a higher life expectancy than males, regardless of their educational attainment, with a gap of more than 5 years globally in each group when analyzing the LE40. However, the analysis for the indicators at age 65 suggests that life expectancy differences for the middle and high education groups were virtually nonexistent, particularly for males.

**Table 2:** Years of life expectancy, CFLE and LEWC (conditional to survival at age 40), separately by cause, sex, and educational attainment (95% CI)

Indicator	Sex	Males			Females		
	Educational attainment	Low	Middle	High	Low	Middle	High
	Life expectancy	40.2	42.7	43.4	46.2	48.5	49.3
CFLE (without condition)	CVD	26.7 (25.8–27.5)	28.7 (28.3–29.1)	30.0 (29.3–30.6)	28.1 (27.2–28.9)	32.4 (32.0–32.8)	34.4 (33.4–35.5)
	Diabetes	33.6 (33.0–34.2)	36.7 (36.4–36.9)	38.3 (37.8–38.8)	37.7 (37.1–38.3)	43.3 (43.0–43.5)	46.7 (46.1–47.3)
	Hypertension	27.6 (26.8–28.5)	28.5 (28.2–28.9)	30.1 (29.5–30.8)	26.8 (26.0–27.7)	31.8 (31.4–32.1)	36.1 (35.1–37.1)
	Mental disorders	33.1 (32.2–33.9)	37.2 (36.9–37.4)	39.7 (39.2–40.1)	30.3 (29.3–31.3)	36.2 (35.8–36.6)	40.0 (39.1–40.9)
	Respiratory conditions	35.6 (35.1–36.2)	39.1 (38.8–39.3)	40.4 (39.9–40.8)	40.8 (40.2–41.4)	44.7 (44.4–44.9)	45.6 (45.0–46.2)
LEWC (with condition)	CVD	13.5 (12.7–14.4)	14.0 (13.6–14.4)	13.3 (12.6–13.9)	18.1 (17.3–19.0)	16.1 (15.7–16.5)	14.9 (13.8–15.9)
	Diabetes	6.6 (6.0–7.2)	6.0 (5.8–6.3)	5.1 (4.6–5.6)	8.5 (7.9–9.1)	5.2 (5.0–5.5)	2.6 (2–3.1)
	Hypertension	12.6 (11.8–13.4)	14.2 (13.8–14.5)	8.4 (7.8–8.9)	19.4 (18.5–20.2)	16.7 (16.4–17.1)	13.2 (12.2–14.2)
	Mental disorders	7.1 (6.3–8.0)	5.5 (5.3–5.8)	3.7 (4.6–5.6)	15.9 (14.9–16.9)	12.3 (11.9–12.7)	9.3 (8.4–10.2)
	Respiratory conditions	4.6 (4.0–5.1)	3.6 (3.4–3.9)	3.0 (2.6–3.4)	5.4 (4.8–6.0)	3.8 (3.6–4.1)	3.7 (3.1–4.3)

Source: author´s calculations based on INE, ENSE and EHIS

**Table 3:** Years of life expectancy, CFLE and LEWC (conditional to survival at age 65), separately by cause, sex, and educational attainment (95% CI)

Indicator	Sex	Males			Females		
	Educational attainment	Low	Middle	High	Low	Middle	High
	Life expectancy	18.9	20.6	20.5	23.1	25.2	25.6
CFLE (without condition)	CVD	10.3 (9.9–10.7)	11.6 (11.2–11.9)	12 (11.3–12.6)	11.4 (11.1–11.8)	13.9 (13.6–14.2)	15 (14.0–16.0)
	Diabetes	14.5 (14.1–14.8)	16.2 (16.0–16.5)	16.6 (16.0–17.1)	17.4 (17.1–17.7)	21 (20.8–21.3)	23.6 (23.0–24.1)
	Hypertension	10.4 (10.0–10.8)	10.8 (10.4–11.1)	11.8 (11.1–12.4)	9.8 (9.4–10.1)	12.4 (12.0–12.7)	15 (14.0–16.1)
	Mental disorders	16.1 (15.9–16.4)	17.8 (17.5–18)	18.5 (18.1–18.9)	15.2 (14.9–15.5)	18.4 (18.1–18.7)	20 (19.1–20.9)
	Respiratory conditions	15.6 (15.3–15.9)	17.9 (17.6–18.1)	18.4 (18.0–18.9)	20.2 (20.0–20.5)	22.8 (22.6–23.0)	23.2 (22.6–23.8)
LEWC (with condition)	CVD	8.6 (8.2–9.0)	9 (8.7–9.3)	8.5 (7.9–9.2)	11.7 (11.4–12.1)	11.3 (11.0–11.7)	10.6 (9.6–11.6)
	Diabetes	4.4 (4.1–4.8)	4.3 (4.1–4.6)	3.9 (3.4–4.5)	5.8 (5.4–6.1)	4.2 (3.9–4.4)	2.0 (1.5–2.6)
	Hypertension	8.5 (8.1–8.9)	9.8 (9.5–10.1)	8.7 (8.1–9.4)	13.4 (13.0–13.7)	12.9 (12.5–13.2)	10.6 (9.5–11.6)
	Mental disorders	2.7 (2.5–3.0)	2.8 (2.5–3.0)	2 (1.6–2.4)	8 (7.6–8.3)	6.8 (6.5–7.1)	5.6 (4.7–6.5)
	Respiratory conditions	2.7 (2.5–2.9)	2.7 (2.5–2.9)	2.1 (1.6–2.5)	2.9 (2.7–3.2)	2.4 (2.2–2.6)	2.4 (1.8–3.0)

Source: author’s calculations based on INE, ENSE and EHIS

In absolute terms, higher-educated individuals presented a higher CFLE at age 40 and 65 in all conditions. Complementarily, they also presented a lower LEWC at age 40 and 65. And the opposite can be said for the low-educated individuals: they had lower CFLEs by each group of causes and higher LEWCs at age 40, for males and females. We can also see that for some conditions, such as CVD or Respiratory, males had a lower CFLE than females. In some conditions, such as hypertension and mental disorders, males with lower education had a slightly higher CFLE than their female counterparts; and females with high education presented values comparable to those of males with high education, but given their longer lifespan, their LEWC was almost twice as high.

For CVD, mental disorders, diabetes, and respiratory conditions, the middle-educated presented a higher or similar LEWCs than their low-educated counterparts. This implies that despite their longer CFLEs, the middle-educated also live longer lifespans with these conditions than their low-educated male counterparts. For females, the LEWC for the middle and low-educated groups overlapped their confidence intervals for some conditions: CVD and respiratory.

### Decomposition of educational differences in CFLE and LEWC at age 40 and age 65

Top half of Table 4 shows for each condition the overall contribution of mortality and morbidity (and the sum of both) in the CFLE at age 40 gaps by education, while the bottom half shows it for the LEWC gaps. Complementarily, Table 5 does the same for the CFLE/LEWC gap at age 65. Note that while the life table data used for each decomposition are the same, the area of the mortality component does need not be the same for each condition, as we are decomposing weighted differences. Results can be interpreted as follows: for instance, the total CFLE educational gap at age 40 of 2.03 years observed between the middle and low-educated (M–L) males for CVD was the sum of 1.4 years of the contribution of a larger lifespan (mortality) and 0.63 years due to the contribution of a lower prevalence (morbidity). Therefore, a positive contribution of both components indicates that the CFLE of the reference group (in the example, middle-educated individuals) reflected advantages in both components: they live longer and healthier (considering this particular group of conditions).

**Table 4:** Contribution of mortality and morbidity into overall differentials in CFLE and LEWC at age 40 by sex and educational attainment, Spain, 2012–2017

Indicator	Condition	Sex	Males			Females		
		Component	Middle–low	High–middle	High–low	Middle–low	High–middle	High–low
CFLE (without condition)	CVD	Morbidity	0.63	0.87	1.50	3.08	1.58	4.66
		Mortality	1.40	0.42	1.82	1.25	0.45	1.71
		All	2.03	1.29	3.32	4.34	2.03	6.37
	Diabetes	Morbidity	1.12	1.06	2.18	3.69	2.82	6.51
		Mortality	1.96	0.55	2.51	1.86	0.67	2.53
		All	3.08	1.62	4.70	5.55	3.49	9.04
	Hypertension	Morbidity	− 0.46	1.19	0.73	3.90	3.90	7.80
		Mortality	1.37	0.4	1.77	1.04	0.42	1.46
		All	0.91	1.58	2.49	4.94	4.32	9.26
	Mental disorders	Morbidity	1.98	1.88	3.86	4.28	3.23	7.51
		Mortality	2.11	0.60	2.71	1.65	0.58	2.23
		All	4.09	2.48	6.57	5.92	3.81	9.73
	Respiratory conditions	Morbidity	1.36	0.66	2.02	1.78	0.27	2.05
		Mortality	2.09	0.62	2.71	2.07	0.68	2.75
		All	3.44	1.29	4.73	3.85	0.95	4.80
LEWC (with condition)	CVD	Morbidity	− 0.63	− 0.87	− 1.54	− 3.08	− 1.60	− 4.68
		Mortality	1.09	0.25	1.34	1.09	0.31	1.40
		All	0.46	− 0.61	− 0.15	− 1.99	− 1.29	− 3.28
	Diabetes	Morbidity	− 1.12	− 1.06	− 2.18	− 3.69	− 2.82	− 6.51
		Mortality	0.52	0.10	0.64	0.48	0.09	0.57
		All	− 0.60	− 0.96	− 1.55	− 3.21	− 2.73	− 5.94
	Hypertension	Morbidity	0.46	− 1.18	− 0.72	− 3.89	− 3.90	− 7.79
		Mortality	1.12	0.27	1.39	1.30	0.33	1.63
		All	1.57	− 0.91	0.66	− 2.59	− 3.57	− 6.16
	Mental disorders	Morbidity	− 1.98	− 1.87	− 3.85	− 4.27	− 3.24	− 7.51
		Mortality	0.39	0.07	0.46	0.69	0.17	0.86
		All	− 1.59	− 1.80	− 3.39	− 3.58	− 3.06	− 6.64
	Respiratory conditions	Morbidity	− 1.4	− 0.67	− 2.02	− 1.78	− 0.26	− 2.04
		Mortality	0.40	0.05	0.45	0.27	0.07	0.34
		All	− 0.96	− 0.62	− 1.58	− 1.51	− 0.20	− 1.71

Source: author’s calculations based on INE, ENSE and EHIS

**Table 5:** Contribution of mortality and morbidity into overall differentials in CFLE and LEWC at age 65 by sex and educational attainment, Spain, 2012–2017

Indicator	Cause	Sex	Males			Females		
		Component	Middle–low	High–middle	High–low	Middle–low	High–middle	High–low
CFLE (without condition)	CVD	Morbidity	0.36	0.44	0.8	1.38	0.89	2.27
		Mortality	0.89	− 0.03	0.86	1.09	0.22	1.31
		All	1.25	0.41	1.66	2.47	1.11	3.58
	Diabetes	Morbidity	0.43	0.38	0.81	2.02	2.17	4.19
		Mortality	1.31	− 0.05	1.26	1.63	0.34	1.97
		All	1.74	0.33	2.07	3.65	2.51	6.16
	Hypertension	Morbidity	− 0.48	1.04	0.56	1.71	2.50	4.21
		Mortality	0.88	− 0.04	0.84	0.88	0.19	1.07
		All	0.4	1	1.4	2.59	2.69	5.28
	Mental disorders	Morbidity	0.24	0.74	0.98	1.75	1.30	3.05
		Mortality	1.4	− 0.04	1.36	1.45	0.29	1.74
		All	1.64	0.7	2.34	3.2	1.59	4.79
	Respiratory conditions	Morbidity	0.89	0.6	1.49	0.79	0.03	0.82
		Mortality	1.37	− 0.03	1.34	1.81	0.35	2.16
		All	2.26	0.57	2.83	2.6	0.38	2.98
LEWC (with condition)	CVD	Morbidity	− 0.35	− 0.43	− 0.78	− 1.38	− 0.89	− 2.27
		Mortality	0.77	− 0.03	0.74	0.97	0.17	1.14
		All	0.42	− 0.46	− 0.04	− 0.41	− 0.72	− 1.13
	Diabetes	Morbidity	− 0.43	− 0.4	− 0.81	− 2.02	− 2.17	− 4.19
		Mortality	0.36	0.0	0.36	0.43	0.05	0.48
		All	− 0.07	− 0.38	− 0.45	− 1.59	− 2.12	− 3.71
	Hypertension	Morbidity	0.48	− 1.03	− 0.55	− 1.71	− 2.50	− 4.21
		Mortality	0.79	− 0.01	0.78	1.17	0.20	1.37
		All	1.27	− 1.04	0.23	− 0.54	− 2.3	− 2.84
	Mental disorders	Morbidity	− 0.24	− 0.74	− 0.98	− 1.75	− 1.31	− 3.06
		Mortality	0.27	− 0.02	0.25	0.60	0.10	0.7
		All	0.03	− 0.76	− 0.73	− 1.15	− 1.21	− 2.36
	Respiratory conditions	Morbidity	− 0.88	− 0.60	− 1.48	− 0.80	− 0.03	− 0.83
		Mortality	0.31	− 0.02	0.29	0.25	0.03	0.28
		All	− 0.57	− 0.62	− 1.19	− 0.55	0	− 0.55

Source: author’s calculations based on INE, ENSE and EHIS

CFLE differences between middle and low-educated males at age 40 showed a greater contribution of the mortality component in all conditions. This mortality gap was larger than the one found for the difference between high and middle-educated males. On the contrary, for these educational groups, CFLE differences were mostly due to the contribution of morbidity, which was larger than the contribution of mortality. Diabetes and mental disorders presented some of the largest educational gaps in CFLE at all educational levels for males, and in these conditions, the morbidity component presented its largest specific contributions.

In contrast, for females the morbidity component was the main contributor to CFLE differences between the middle and low-educated for all groups of conditions except respiratory conditions. Similarly, the contribution of the morbidity component was also the main driver of differences between the high and middle-educated groups of females. When it comes to the differences in LEWC among groups, in most cases the mortality component is smaller in magnitude than the morbidity component, meaning that morbidity explains to a larger extent the lower LEWCs of more educated individuals.

This implies that if mortality had been the same for all groups, the health gaps by education would have been even larger than originally estimated, for both males and females and particularly, for the gap between the middle and low-educated groups, where the contribution of mortality was more important. Thus, mortality masked part of the observed LEWC health gap. The only exceptions are the educational gaps between the middle and low education groups among males for CVD and hypertension, where the total difference in LEWC was positive (0.46 and 1.57 years, respectively). In these cases, middle-educated males (the reference group in this scenario) lived more years with these conditions than low-educated males, and the decomposition analysis reveals that the mortality advantage was the component that is the main cause of this.

Complementarily, Table 5 shows the corresponding contribution of mortality and morbidity for the CFLE/LEWC gap at age 65. When comparing the low and middle education group among males, we can see that mortality was the main driver of the CFLE gaps, while the contribution of morbidity explained only a small part of the difference (in respiratory conditions the contribution of the morbidity seems to be the largest of all conditions, and in hypertension the contribution of morbidity is negative, implying a higher prevalence of the middle-educated group after age 65). For females, when addressing the gap between the middle and low education group, both morbidity and mortality presented an important contribution on the differentials: however, for respiratory conditions, unlike the four remaining conditions, mortality presented a largest contribution to the CFLE gap.

When addressing the CFLE gap between high and middle-educated males, the mortality component virtually vanished (which was something we could expect due to the mortality convergence between those groups), meaning that the contribution of morbidity explained the difference across those groups. This was the case for females as well, broadly speaking. However, mortality presented a small positive contribution to the CFLE gap, and in the case of respiratory conditions, it is responsible for almost the entirety of the gap since the contribution of morbidity was minimal for that group of conditions. For the LEWC gaps at age 65 between low and middle groups, the contribution of mortality, just like for the gaps at age 40, mitigates part of the differences for males and females, implying that differences among groups would have been larger if not for the mortality contribution (except for hypertension among males). Between the high and middle education groups, the absence of the mortality component indicates that differences in the indicators are the results of a higher prevalence of the conditions among groups, and not from a higher longevity.

**Fig. 3:**
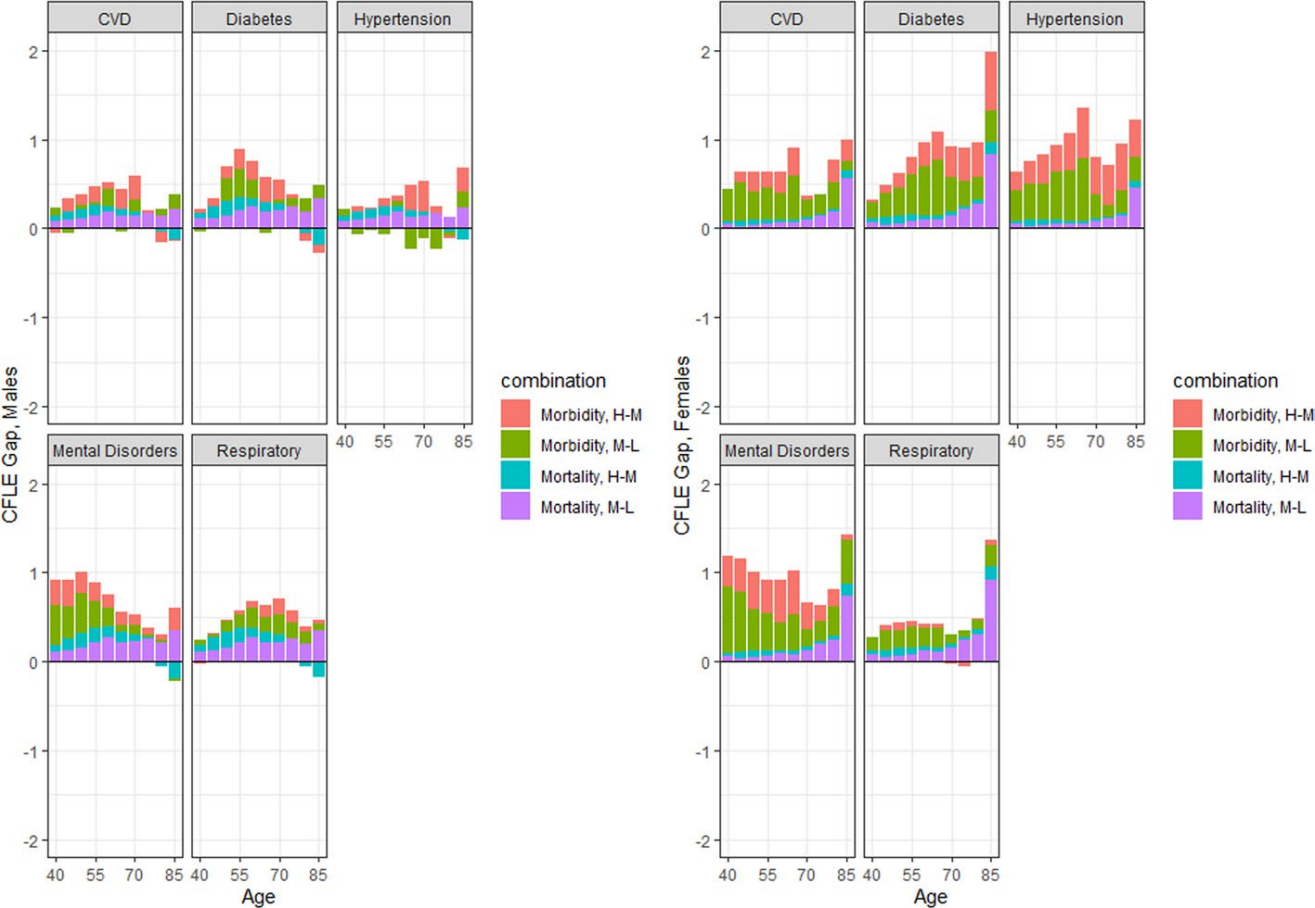
Age-specific contribution of mortality and morbidity into overall differentials in CFLE at age 40 by sex and educational attainment, Spain, 2012–2017 (Source: author's calculations based on INE, ENSE and EHIS. Key: H-M: High minus Middle. M-L: Middle minus Low)

**Fig. 4:**
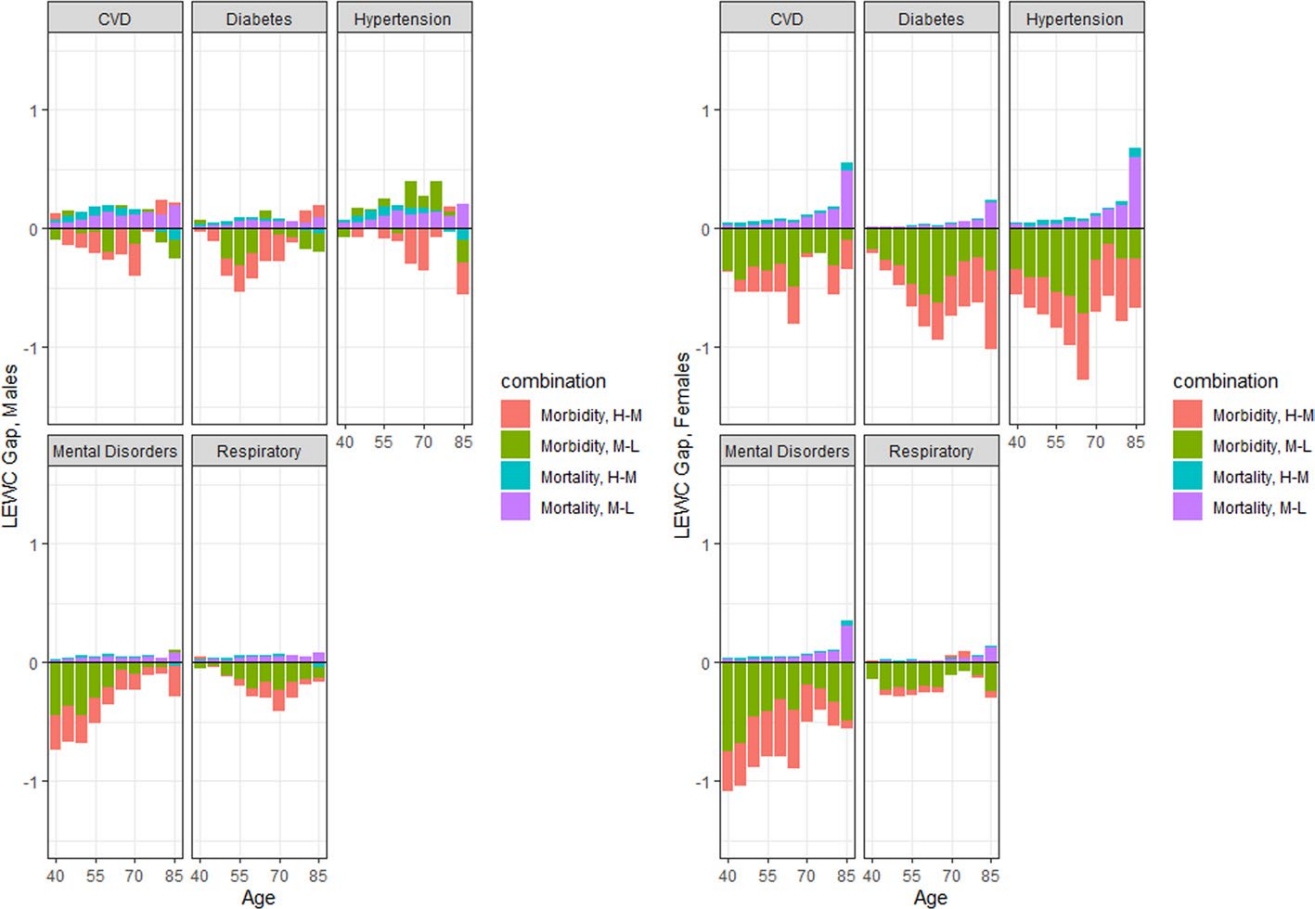
Age-specific contribution of mortality and morbidity into overall differentials in LEWC at age 40 by sex and educational attainment, Spain, 2012–2017 (Source: author’s calculations based on INE, ENSE and EHIS Key: H-M: High minus Middle. M-L: Middle minus Low)

Figures 3 and 4 display the age-specific contributions to the CFLE and LEWC differentials at age 40, respectively. In this case, the sum of the two partial differences is equal to the total gap between the high and low education groups (in other words, the total bar height equals the difference between the high and low education groups).

The left half of Fig. 3 shows the age-specific decomposition for males. There, we can observe the contribution of morbidity particularly in mental disorders and diabetes, where the CFLE gaps are larger, and specially in the younger age groups. For all conditions, the mortality gradients seem to be more evenly distributed across the life course, although we can observe a sustained increase in the contribution of the mortality gap between middle and low-educated males, and nearing age 65, the contribution of mortality between the high-educated and the middle-educated group becomes negligible when addressing the age-specific contribution.

The right half of Fig. 4 presents the age-specific decomposition for females. The age-specific CFLE gap between the high and the middle-educated groups varies by condition, with a high presence in the younger age groups for mental disorders, a larger contribution in the oldest age groups for diabetes, and a larger presence in the middle age groups for hypertension. The contribution of morbidity is also more present in the younger and middle age groups in most conditions, particularly in the gap between the middle and low-educated groups: just as for males, the contribution of mortality after age 65 between the high and middle-educated groups is almost nonexistent. However, the exception was the final age group, which has the largest contributions of mortality in the two group differences, (particularly between the difference in the middle minus low-educated group) given the fact that there is still a fair share of females who are still alive at age 85.

Figure 4 shows the age decomposition of the LEWC educational differences. As mentioned above, the contribution of the morbidity component is the opposite of its CFLE counterpart, so we focus on the analysis of the mortality components. Among males, the mortality contribution was positive and larger for the gap between those with middle and low education. Regarding age patterns, no clear age pattern was found for the small mortality differentials for most chronic conditions. For females, most of the mortality differentials in the LEWC were concentrated in the oldest age groups (suggesting that the gap in LEWC in earlier age groups was due to morbidity differences alone), mostly driven by the gap between those with middle and low education.

## Discussion and final comments

In this study, we investigated the contribution of mortality and morbidity components to differences in CFLE (condition-free life expectancy) and LEWC (life expectancy with the condition) at age 40 and 65 by educational attainment for males and females in Spain. We analyzed several groups of high-prevalence conditions on health expectancies, identified the morbidity and mortality components responsible for such disparities, and highlighted which age groups were the ones that contributed the most to such differences. This is important because it provides policymakers with valuable information to understand and mitigate the health inequality dynamics behind life expectancy spent in good and in poor health, which may differ across socioeconomic status and by sex.

First of all, we have to mention that health expectancies are generally not independent of mortality dynamics. In the Spanish case, we identified a convergence in death rates between individuals in the middle-educated group and the high-educated group around the age of 70. There are two plausible, albeit speculative, explanations for this convergence. On the one hand, the effects of the educational expansion in Spain might be relatively recent, as getting a university degree was a rather rare event in the past. This might mean that simply post-primary education might represent a similar advantage in health than an advanced college degree after age 65 (Permanyer et al., 2018). On the other hand, cohort effects might be playing a part in mortality convergence, specifically with certain causes of death such as lung cancer. Previous studies have pointed out that cohorts born between 1940 and 1960 were particularly affected by lung cancer due to the expansion of smoking that occurred in Spain since the second half of the twentieth century (Franco et al., 2002; Ocaña-Riola et al., 2013). As was the case for many countries, it is possible that the forerunners of the habit in Spain might have been individuals from a more advantaged economic position before it became widespread in the whole society (Giskes et al., 2005; Graham, 1996). This example, based on cancer, might also have been the case for other causes of death or overall mortality, indicating that in the future the mortality dynamics might shift into a stronger process of convergence if cohort effects do dissipate. Regarding the healthy life expectancy of adults in Spain, we found a clear educational gradient for both males and females and all the health conditions examined (CVD, Diabetes, Hypertension, Mental Disorders, and Respiratory Conditions). However, unhealthy life expectancies did not show such a gradient and results differed by sex and were particularly interesting among males. Middle-educated males had longer life expectancy with CVD, Diabetes, and Respiratory Conditions than those with higher education and, also, with lower levels of education. In addition, several estimates of LEWC for males and females were not much different. This study, by decomposing educational differences in healthy and unhealthy life expectancies for given conditions into the mortality and morbidity components and by age, contributed to providing insights into the main drivers of such educational gaps.

The educational gradient in the prevalence and condition-specific CFLEs was present in all of the conditions analyzed. This is in line with previous research, where educational gradients have been found when analyzing aggregate measures or indicators such as the GALI or indexes in limitations in daily activities (ADL) in Spain (Gumà et al., 2019; Solé-Auró & Alcañiz, 2016; Solé-Auró et al., 2020, 2022).

For males, we identified that differences in CFLE at age 40 between the middle and low-educated groups in Spain are mainly explained by mortality components, while differences between higher and middle-educated groups are mainly explained by morbidity components. This sounds especially true after age 65, where differences in CFLE between those groups were almost entirely attributed to differences in morbidity. In other words, the advantage of more educated males over their less educated counterparts is attributed to both their longevity and healthier lifespans, but depending on their educational position, it can be predominantly due to one component over the other. This also meant that while higher-educated males had the shortest unhealthy lifespans overall, middle-educated individuals lived longer, but on occasion had longer unhealthy life expectancies when compared to the low-educated group because of their lower mortality. This could be indicative, in theory, that the increase in life expectancy for higher-educated males does not translate into an increase in the time lived with conditions, given the lower disease prevalence that goes with such an increase in longevity, but this is not the case for middle-educated males. Furthermore, this is indicative that the contribution of mortality is more relevant at specific age-groups when addressing the health gap by educational attainment. After age 65 the disappearance of the mortality component between high-educated and middle-educated groups suggests that differences in CFLEs are purely the result of difference in prevalences of the analyzed conditions, implying a lower quality of life for the middle-educated group despite having a similar longevity.

For females, morbidity was the main driver of educational differences in CFLE for most conditions (except Respiratory Conditions). Mortality also played a role in the gap between the middle and low-educated groups. For the CFLE gap between the higher and middle-educated groups of females, mortality differences were modest. These results are in line with the findings of other studies that have focused on this issue, albeit with different magnitudes, we can see that the contribution of the mortality component to health expectancy differentials is smaller for females than for males (Nusselder et al., 2005; Yokota et al., 2019). In the Spanish case, this is mainly because educational differences in mortality for females tend to be smaller than for males (Huisman et al., 2005; Reques et al., 2014). Simply put, while for males the CFLE advantage that higher educated individuals had was because of a combination of mortality and morbidity components (implying both longer and healthier life expectancies), for females, the morbidity component was the main determinant of the difference.

The condition-specific analysis showed educational inequalities for all conditions, but greater for hypertension, mental disorders, and diabetes. The clear educational gradient in diabetes, for both males and females, is consistent with the previous evidence showing these disparities in Spain (Reques et al., 2014; Ruiz-Ramos et al., 2006). This is likely due to the adoption of risky behaviors, a greater proportion of obese people among the educated, and poorer food quality consumption. It is also the only condition in which the sex gap in morbidity favors females: apart from their longer lifespans and CFLE, the LEWC for middle and highly educated females is smaller than for males, because of their lower overall prevalence.

In mental disorders we found some of the largest educational gaps in prevalence and in CFLE for both males and females. It is worth mentioning that the higher prevalence in females implied that, despite their longer life expectancy, the years of CFLE were the same for both sexes. However, the age-specific decomposition indicated that most of the morbidity gap by education was concentrated in the younger age groups, especially for males, and before the age of 65. After age 65, mortality only remains a driver of differences in CFLE when comparing to the lower-educated groups. For females, educational differences in morbidity were found in all age groups, and with a high intensity in the younger ones. This is consistent with previous literature stressing the relationship between occupational status, educational attainment, and mental health, which is mostly present during the working age (Bell, 2014; Kessler et al., 2010).

For CVD, we found some of the lowest CFLEs of all our estimations for both males and females, particularly among those with higher education, who had the highest CFLE compared to other conditions in the same level of education. Furthermore, among males, the morbidity gap between levels of education was one of the smallest of all the conditions analyzed, with most of the differences concentrated in the younger and mid-adult ages before converging at older ages.

In hypertension, we found that females had a higher prevalence than males, and as a result, their LEWC is higher despite a similar CFLE. In addition, we found that the educational gap in morbidity is small for males but large for females, is evenly distributed across the life course, and persists into the oldest ages. Note that there may be some underreporting of prevalence due to the way the survey collects information on conditions such as CVD and Hypertension. This may be especially true for lower-educated individuals at younger ages, where routine medical checkups or other forms of detection may be less frequent, and the impact of these two groups of conditions on overall health may not be as obvious as in the case of back pain or mental health conditions (Everett & Zajacova, 2015; Ong et al., 2007; Spitzer, 2020).

Finally, for respiratory conditions, we found the highest CFLE for both males and females and, correspondingly, some of the lowest LEWC. In both cases, most of the (rather modest) differences in morbidity were found between the middle and the lower-educated groups, suggesting that there were only small differences between the higher-educated and the middle educated. The early appearance of respiratory conditions in childhood (Newacheck & Taylor, 1992) and the overall low prevalence of COPD and asthma may explain the lack of differences between educational groups.

While educational and sex disparities in some conditions such as mental health in Spain were documented previously (Balanza Galindo et al., 2009; Cardila et al., 2015; Raya-Tena et al., 2021; Rocha et al., 2015), they were rarely compared with other causes of disease. In this regard, another interesting finding of our study is the larger socioeconomic gaps for some specific groups of conditions when compared to others, and the magnitude of the socioeconomic gradient at certain age groups. The fact that mental disorders presented larger educational disparities in health, may also be related to the fact that lower educated individuals are more at risk of such conditions (due to their exposures across the life course), or are more affected by them in their perceptions of health.

This study was not exempt from limitations, which are openly acknowledged. First of all, even by pooling three health surveys, the relatively small sample size forced us to consider three categories of educational attainment, possibly ignoring more subtle layers of analysis if more categories were used, although arguably facilitating the interpretations of the decomposition analysis. Furthermore, pooling the surveys allowed us to have more robust confidence intervals for our estimates. In addition, this study did not consider the institutionalized population. According to the National Institute of Statistics, this group represented the 1% of the whole population in Spain in 2021. However, more than 3% of males and 5% of females aged 85 and above were living in collective residences such as nursing homes. While we acknowledge that the prevalence of chronic conditions at the final age groups may be somewhat underestimated as a result, this particular subgroup does not seem to be large enough to substantially alter our summary estimations, especially if we consider only individuals aged 40 and over and that there should not be very strong compositional changes in this population, as pointed out by Zueras and Rentería (2020). We have also presented the results at the country level without considering any regional spatial differentiation, although this is something to consider for future studies. It should also be mentioned that although most of these conditions were diagnosed by a doctor, the registered answers are still reliant on the veracity of the respondent, which means that in some conditions there may be some underreporting. This could be the case for hypertension or cardiovascular conditions in males with low education. Furthermore, the ailments that make up the different conditions we grouped together may also introduce their own degree of heterogeneity. However, since we pooled observations into a single large observation, we can somewhat mitigate large differences in prevalence within groups.

Most importantly, the methodological approach has its shortcomings. The Sullivan method relies on the assumption that morbidity and mortality rates are subject to the same life table, and in this case, we must assume that individuals with different health conditions are subject to a similar lethality by education. However, since Spain is a country with overall low levels of amenable mortality, we believe that such an assumption should not sensibly alter our estimates. Previous studies (e.g., Imai and Soneji, 2007, Mathers and Robine, 1997) have shown that in the absence of abrupt changes in population health, the estimates produced by the Sullivan method are similar to those produced by other techniques that require longitudinal or repeated cross-sectional data (e.g., Guillot and Yu, 2009). Furthermore, since the Sullivan method is still widely used in research (e.g., Canudas-Romo et al., 2017; di Lego et al., 2020; Solé-Auró et al., 2022; Yokota et al., 2019), we believe that it is a valid option for estimating health expectancies while acknowledging these limitations.

Despite such limitations, we believe that the findings presented in this paper prove the advantages of considering chronic conditions separately when investigating the health burden in a population, particularly by identifying the mechanisms behind the differences in condition-specific health expectancies by educational attainment in Spain.

These results suggest that aggregate measures (i.e., health expectancies based on the presence of one or more conditions in the same measure) mask certain heterogeneities in health in Spain (Voigt et al., 2020). Additionally, it brings a word of warning, because grouping categories of chronic conditions and global indicators of disability could lead to an underestimation of educational disparities in health indicators. Possibly, separating mental disorders from physical conditions could be an interesting approach to visualize social inequalities in population health when analyzing health inequalities in the future.

More importantly, the results suggest that the gaps in morbidity play a more prominent role in explaining differences in health expectancies for many conditions as mortality tends to converge across populations, given the rather small magnitude of differences in life expectancy in Spain. In other words, if differences in mortality tend to decrease in the future, we should expect morbidity to be the main driver of overall differences in health expectancies. Therefore, adequate monitoring of the health burden of each chronic condition separately would provide a more accurate picture of social inequalities in health and allow for the design of adequate policies to mitigate or adapt to them.

## Data Availability

The code and data for this manuscript can be provided upon reasonable request.

## Appendix

See Table 6.

**Table 6:** Classification of chronic conditions based on ENSE and EHIS Questionnaires

Group	Condition	Questions in ENSE 2012	Questions in	Questions in
			ENSE 2017	EHIS 2014
Cardiovascular diseases	Myocardial infarction	G21b-2, G21c-2	G25b-2, G25c-2	G25b-2, G25c-2
	Stroke	G21b-22, G21c-22	G21b-23, G21c-23	G21b-23, G21c-23
	Other heart diseases	G21b-3, G21c-3	G25b-3, G25c-3	G25b-3, G25c-3
				G25c-4, G25-c4
	High cholesterol	G21b-14, G21c-14	G25b-15, G25c-15	G25b-15, G25c-15
Respiratory conditions	COPLD	G21b-10, G21c-10	G25b-11, G25c-11	G25b-11, G25c-11
	Asthma	G21b-9, G21c-9	G25b-10, G25c-10	G25b-10, G25c-10
Diabetes	Diabetes	G21b-11, G21c-11	G25b-12, G25c-12	G25b-12, G25c-12
Hypertension	Hypertension	G21b-1, G21c-1	G25b-1, G25c-1	G25b-1, G25c-1
Mental disorders	Depression	G21b-19, G21c-19	G25b-20, G25c-20	G25b-20, G25c-20
	Anxiety	G21b-20, G21c-20	G25b-21, G25c-21	G25b-21, G25c-21
	Other mental disorders	G21b-21, G21c-21	G25b-22, G25c-22	G25b-22, G25c-22

Source: author’s calculations based on ENSE and EHIS

## Abbreviations

CFLE: Condition-free life expectancy
LEWC: Life expectancy with conditions
LE: Life expectancy
ENSE: Encuesta Nacional de Salud de España (Spanish National Health Survey)
EHIS: European Health Interview Survey
CVD: Cardiovascular conditions.
M-L: Middle Education minus Low Education
H-M: High Education minus Middle Education
